# Insights into transcriptional changes that accompany organelle sequestration from the stolen nucleus of *Mesodinium rubrum*

**DOI:** 10.1186/s12864-015-2052-9

**Published:** 2015-10-16

**Authors:** Erica Lasek-Nesselquist, Jennifer H. Wisecaver, Jeremiah D. Hackett, Matthew D. Johnson

**Affiliations:** University of Scranton, 800 Linden St., Scranton, PA 18510 USA; Vanderbilt University, VU Station B 351364, Nashville, TN 37235 USA; University of Arizona, Tucson, AZ 85721 USA; Woods Hole Oceanographic Institution, 266 Woods Hole Road, Woods Hole, MA 02543 USA

**Keywords:** *Mesodinium rubrum*, *Geminigera cryophila*, Karyoklepty, Acquired phototrophy, Transcriptome, Differential gene expression, Chimeric metabolism, Organelle retention, Mixotrophy

## Abstract

**Background:**

Organelle retention is a form of mixotrophy that allows organisms to reap metabolic benefits similar to those of photoautotrophs through capture of algal prey and sequestration of their plastids. *Mesodinium rubrum* is an abundant and broadly distributed photosynthetic marine ciliate that steals organelles from cryptophyte algae, such as *Geminigera cryophila. M. rubrum* is unique from most other acquired phototrophs because it also steals a functional nucleus that facilitates genetic control of sequestered plastids and other organelles. We analyzed changes in *G. cryophila* nuclear gene expression and transcript abundance after its incorporation into the cellular architecture of *M. rubrum* as an initial step towards understanding this complex system.

**Methods:**

We compared Illumina-generated transcriptomes of the cryptophyte Geminigera cryophila as a free-living cell and as a sequestered nucleus in M. rubrum to identify changes in protein abundance and gene expression. After KEGG annotation, proteins were clustered by functional categories, which were evaluated for over- or under-representation in the sequestered nucleus. Similarly, coding sequences were grouped by KEGG categories/pathways, which were then evaluated for over- or under-expression via read count strategies.

**Results:**

At the time of sampling, the global transcriptome of *M. rubrum* was dominated (~58–62 %) by transcription from its stolen nucleus. A comparison of transcriptomes from free-living *G. cryophila* cells to those of the sequestered nucleus revealed a decrease in gene expression and transcript abundance for most functional protein categories within the ciliate. However, genes coding for proteins involved in photosynthesis, oxidative stress reduction, and several other metabolic pathways revealed striking exceptions to this general decline.

**Conclusions:**

Major changes in *G. cryophila* transcript expression after sequestration by *M. rubrum* and the ciliate’s success as a photoautotroph imply some level of control or gene regulation by the ciliate and at the very least reflect a degree of coordination between host and foreign organelles. Intriguingly, cryptophyte genes involved in protein transport are significantly under-expressed in *M. rubrum*, implicating a role for the ciliate’s endomembrane system in targeting cryptophyte proteins to plastid complexes. Collectively, this initial portrait of an acquired transcriptome within a dynamic and ecologically successful ciliate highlights the remarkable cellular and metabolic chimerism of this system.

**Electronic supplementary material:**

The online version of this article (doi:10.1186/s12864-015-2052-9) contains supplementary material, which is available to authorized users.

## Background

**Fig. 1:**
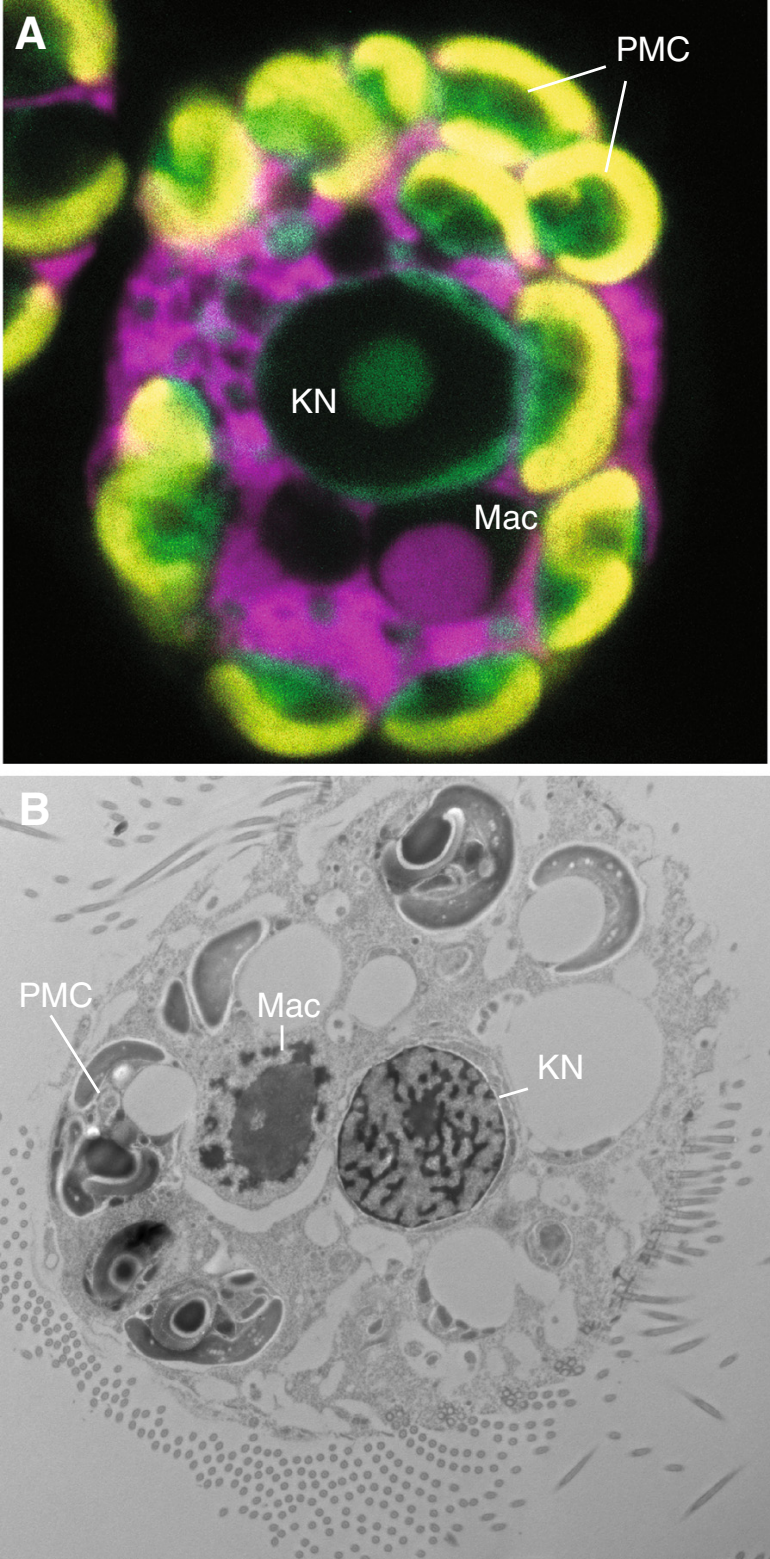
*Mesodinium rubrum* and its foreign organelles. **a** fluorescence micrograph showing results from fluorescence in situ hybridization using dual probes for the *M. rubrum* (*pink*) and *Geminigera cryophila* (*green*) small subunit rRNA genes as described by Johnson et al. (2007). Plastid fluorescence appears yellow. **b** Transmission electron micrograph image of *M. rubrum* as described in Johnson et al. (2006). PMC, plastid-mitochondrial complex; KN, kleptokaryon (cryptophyte nucleus); Mac, ciliate macronucleus

The temporary acquisition of phototrophy by hosting algal endosymbionts or retaining functional plastids from algal prey are widespread phenomena in aquatic ecosystems and at certain times can make important contributions to community productivity [1, 2]. *Mesodinium rubrum* is a globally distributed marine and estuarine mixotrophic ciliate with fully functional acquired cryptophyte organelles that are maintained in a symbiotic-like state (Fig. 1) [3–7]. While these foreign organelles can divide in the ciliate, they are not stable components of the cell and there is no evidence that *M. rubrum* possesses the genetic machinery necessary to control them. Rather, the ciliate steals the nucleus from cryptophyte prey, a process described as karyoklepty (Fig. 1) [6]. The nucleus can remain active for over 2 weeks but does not appear to undergo karyokinesis. During this time, nucleus-encoded plastid-targeted genes are expressed and pigment synthesis, plastid division, and cell division occur at their maximum rates [6, 8]. Once the stolen nucleus is lost, chloroplast division ceases, and cell division steadily drops over time [6]. While there is strong evidence that karyoklepty facilitates exploitation of prey organelles, the extent to which the kleptokaryon remains active and contributes to maintaining sequestered organelles is unknown.

*Mesodinium rubrum* is one of the most common and abundant organelle-retaining protists [1] but its use of a prey nucleus and its reliance upon phototrophy is atypical. Strikingly, most (>90 %) of the ciliate’s C budget derives from photosynthesis [8, 9] and it utilizes nitrate [10, 11]. Oligotrich ciliates and most other organelle-retaining protists are more mixotrophic in their metabolism and predominantly employ phagotrophic heterotrophy for their growth needs while products of photosynthesis predominantly satisfy respiration needs [12, 13].

In contrast to *M. rubrum*, plastids in oligotrich ciliates are relatively short-lived and do not divide [14, 15]. A recent study revealed similar transcriptomic repertoires for the plastid-sequestering oligotrich ciliate, *Strombidium rassoulzadegani* and its close heterotrophic relative, *Strombidinopsis* sp., [16]. However, *S. rassoulzadegani* had a greater complement of genes participating in oxidative stress responses than *Strombidinopsis* sp., which may be an adaptation to harboring plastids [16]. *Paramecium bursaria* grown with and without *Chlorella* algal symbionts also displayed differential expression of genes that mitigate oxidative stress [17]. Ciliates grown with their symbionts decreased the expression of glutathione S-transferase [17], which suggests that the additional protection against reactive oxidative species in this system stems from concomitant changes in gene expression in *Chlorella*. Other down-regulated genes in the host included those potentially involved in fatty acid and sugar production [17]. Thus, *Chlorella* might reduce the role the host plays in carbon metabolism. Because, *M. rubrum* maintains a kleptokaryon that actively participates in maintaining and dividing stolen plastids, we predict that transcription from the kleptokaryon will be highly active.

Here we compare the transcriptome of the cryptophyte *Geminigera cryophila*, both as a free-living cell and as a sequestered nucleus in *M. rubrum*. This initial analysis provides insight into the molecular dynamics associated with temporary organelle integration within a host cell and highlights pathways to examine in *M. rubrum* that might demonstrate compensatory changes or communication between the host and kleptokaryon. While only an initial snapshot of a complicated system, our research supports the idea that foreign organelles heavily influence the molecular landscape of *Mesodinium rubrum* during certain points in its life cycle. Continued investigations into the dynamics of host-sequestered organelle interactions throughout the life cycle of the ciliate will provide a more general overview of molecular integration.

## Methods

### Cultures, sampling, and RNA extraction

An Antarctic strain of the ciliate, *Mesodinium rubrum* (CCMP 2563) was grown at 4 °C in F/2 (−Si) medium at 200 nmol photonsm^−2^ s^−2^ (14 h light:10 h dark) under cool white fluorescent lights in 31 PSU seawater at pH 8.2. At time of sampling, the batch culture was at 20,000 cells per mL, and had been fed approximately 3 weeks earlier with *Geminigera cryophila* (CCMP 2564) at a ratio of 1 prey/1predator, with only trace levels of the prey remaining (<1000 cells per mL). This ensured that free-living *G. cryophila* cells not ingested by *M. rubrum* made minimal contributions to the transcriptome of the ciliate. Previous research on this strain has demonstrated that M. rubrum is capable of clearing cryptophyte prey at even higher prey/predator ratios within 1 week [8]. Thus, any transcripts of cryptophyte origin identified in the *M. rubrum* transcriptome were assumed to derive from the sequestered nucleus of *G. cryophila* and these sequences were compared against transcripts from free-living *G. cryophila* cells. Free-living *Geminigera cryophila* (CCMP 2564) cultures were grown under the same conditions as *M. rubrum*. RNA was extracted via a standard Trizol procedure and sent to the National Center for Genome Research (Santa Fe, NM) for cDNA synthesis and sequence analysis.

### Sequencing and transcriptome assembly

Sequence data for this study were prepared through the Marine Microbial Eukaryote Transcriptome Project (MMETSP) [18]. Fastq files of pre-processed reads as well as Fasta files of coding and protein sequences, and transcriptome assemblies are available through iMicrobe commons (data.imicrobe.us), an interactive data commons. Data generated by the MMETSP project are also accessible in the sequence read archive (SRA) of NCBI under the BioProject PRJNA248394. We analyzed Fastq files of pre-processed reads as well as coding and protein sequences from the transcriptomes of *G. cryophila* (sample name MMETSP0799; SRA accession SRS618815) and *M. rubrum* (*Myrionecta rubra*; sample name MMETSP0798; SRA accession SRS618816). Sequencing of RNA Tru-Seq libraries was performed on an Illumina HiSeq 2000 platform followed by de novo assembly. Sequencing procedures and assembly pipeline are outlined on the MMETSP website (http://marinemicroeukaryotes.org/home/faq). Briefly, the assembly pipeline involved a preprocessing step to remove Illumina primers, adaptors, and PhiX174 control DNA and a quality trimming step (>Q15) using the FASTX-toolkit (http://hannonlab.cshl.edu/fastx_toolkit). ABySS v.1.3.2 [19] performed *de novo* assembly and scaffolding, CAP3 [20] generated a consensus assembly of contigs, and GapCloser v1.10 from SOAP *de novo* [21] resolved gaps. ESTscan [22] identified potential coding sequences and HMMER v.3.1b1 (http://hmmer.janelia.org) searches against PFAM (http://pfam.sanger.ac.uk) and TIGRFAM (http://www.jcvi.org/cgi-bin/tigrfams/index.cgi) databases as well as BLASTP searches [23] against the SwissProt database (www.uniprot.org) provided protein annotations.

We mapped reads back to all *M. rubrum* and *G. cryophila* coding sequences (11,893 and 44,315, respectively) with Bowtie v.1.1.1 [24] using default parameters. SAM files from Bowtie were converted to indexed, sorted BAM files with SAMtools v.0.1.17 [25] and BAM files were converted to bed files with the Python (www.python.org) script “make_bed_from_fasta.py,” from the Angus 2.0 website (http://ged.msu.edu/angus/tutorials-2013/rnaseq_bwa_counting.html). The multiBamConv subroutine, which is part of the BEDTools package v.2.20.1 [26], then counted paired reads with a quality value of 30 or greater that mapped to each gene.

### Database filtering

We analyzed transcriptomic changes that occurred in the sequestered nucleus of *G. cryophila* (herein referred to as KN) as compared to free-living *G. cryophila* cells (herein referred to as GC) as an initial step towards understanding the interactions between host and prey organelles. Because we lack reference genomes for *Geminigera cryophila* and *Mesodinium rubrum*, BLASTP searches against a customized reference database separated KN sequences from those of *M. rubrum*. The database, generously provided by Woehle et al. (2011) [27], included proteomes from all eukaryotic supergroups (i.e. microbial eukaryotes) with additional sequences from the cryptophyte proteome of *Guillardia theta*, several proteomes from Cyanobacteria and Streptophytes, and protein sequences derived from the free-living *G. cryophila* transcriptome (MMETSP0799). Although the free-living *G. cryophila* transcriptome does not necessarily represent the expression of an entire proteome, the inclusion of these sequences should improve our ability to identify KN proteins within the *M. rubrum* dataset. Sequences with a top BLAST hit to any cryptophyte with an e-value of 1 × 10^−4^ or better were designated as KN. Sequences with top BLAST hits to non-cryptophyte species could represent sequences of either *M. rubrum* or KN origin but we tentatively designated them as *M. rubrum*. At the time these filtering strategies were employed, only two distantly related ciliates to *M. rubrum*—*Tetrahymena thermophila* and *Paramecium bursaria*—had sequenced genomes. Thus, ciliates are not well represented in our reference database. Given the paucity of ciliate sequences available and the fact that *Mesodinium* shows rapid sequence evolution [28], it is likely that *M. rubrum* proteins will return best BLAST hits to non-ciliate species.

### Functional annotation and evaluation of pathway differences between KN and GC

The KEGG automated annotation server (http://www.genome.jp/tools/kaas/) [29]—with the unidirectional best hit and EST annotation options—assigned functional annotation to the coding sequences in the GC and KN datasets. The unidirectional best BLAST hits option was chosen to maximize the number of proteins annotated because *Geminigera cryophila* and *Mesodinium rubrum* lack reference genomes and microbial eukaryotes with secondary red plastids (such as cryptophytes) have minimal representation in the KEGG database. Chi square tests in R v.3.1.2 (http://www.R-project.org) identified significant differences in protein abundance levels between GC and KN for various KEGG categories/pathways (herein these analyses will be referred to as protein count analyses). A second method for evaluating changes in category or pathway prevalence relied on read counts that mapped to coding sequences. We determined the number of reads mapping to each gene with Bowtie, normalized read count by gene length, and tallied the number of reads per base pair for each gene in each functional category/pathway to obtain a proxy for the total amount of expression. Mann–Whitney tests performed using a custom-designed Python v.2.7.5 script determined whether categories/pathways showed significant over or under expression in GC versus KN (herein these analyses will be referred to as read count analyses). Protein count analyses were performed for the subcategories directly under the following broader categories: 1. Metabolism, 2. Genetic information and processing, 3. Environmental information and processing, and 4. Cellular processes (Additional file 1: Table S1). Gene expression analyses were performed at this sub-category level and for more specific pathways within each of these sub-categories (Additional file 1: Table S1). The p.adjust function in R adjusted *P*-values to a false discovery rate of < 0.05 [30] for protein and read count tests.

### DESeq and edgeR analyses of differential gene expression

Although we lacked biological replicates for this experiment, we employed DESeq v.1.11.6 [31] and edgeR v.3.4.2 [32] in the Bioconductor package [33] of R to determine whether results from these tools would be consistent with or reveal the same trends as those obtained from read count analyses. Additionally, we focused on log2fold changes (log2FCs) between genes in GC and KN rather than statistically significant results to avoid overreaching conclusions. BLASTP searches identified reciprocal best blast hits (RBBHs) between KN and GC datasets. These RBBH genes were then subject to DESeq and edgeR analyses. In DESeq, the estimateSizeFactors command normalized datasets. Variance was estimated with the “blind” method and the additional parameters “fit-only” and “local” were employed for sharingMode and fitType, respectively. In edgeR, the TMM method [34] normalized dataset sizes for GC and KN. In an attempt to account for the effects of biological variability within our data, we chose a dispersion value of 0.3, which is between the recommended values as outlined in the edgeR user’s guide [35] and applied exact negative binomial tests to identify differentially expressed genes.

## Results

### Filtering and annotation

BLASTP searches identified 7782 polypeptide sequences as putatively of *Geminigera cryophila* (KN) origin out of a total of 12,650 proteins called for the *Mesodinium rubrum* transcriptome. This indicates that at least when this culture was sampled, sequences from the KN heavily dominated (61.5 %) the composition of the “global” *M. rubrum* transcriptome. Applying a more stringent cutoff to identifying KN sequences, where only proteins returning a cryptophyte hit with an *e*-value of 1 × 10^−30^ or better were considered of KN origin, still resulted in 58 % of the *M. rubrum* transcriptome as being derived from the stolen nucleus. Conversely, applying a more stringent criterion for assigning proteins to *M. rubrum* proportionally increased the contribution of KN to the transcriptome. For example, considering only proteins with best BLAST hits (*e*-value of 1 × 10^−4^ or better) to ciliate sequences in our reference database yielded only 271 proteins of *M. rubrum* origin. The average coding sequence length was ~850 +/− 648 bp and the average GC content was 55 +/− 0.038 %, which corresponds well with the average GC content calculated for the free-living *G. cryophila* transcriptome (55 +/− 0.041 %). We submitted the entire GC dataset of 45,232 proteins (average length 1100 +/− 1134 bp) and the 7782 putative KN sequences to KEGG for annotation, which assigned KO (KEGG orthology group) numbers to 7184 GC and 2657 KN polypeptide sequences, respectively.

### Protein count results

Amino acid, carbohydrate, and energy metabolism categories showed statistically significant overrepresentation for the KN dataset in comparison to the GC (Table 1). Proteins related to transcription, translation, and metabolism of other amino acids also showed a significant increase in KN (Table 1). Proteins within categories predominantly related to cellular functions were significantly less abundant in KN compared to GC (Table 1).

**Table 1:** Protein count analyses for select KEGG subcategories

Category	Number of proteins		% of total		*P*-value adjusted
	GC	KN	GC	KN	
**Amino acid metabolism**	374	242	7	10	1E-04
**Carbohydrate metabolism**	441	249	9	11	1E-02
**Cell communication**	152	38	3	2	5E-03
**Cell growth and death**	302	69	6	3	5E-06
**Cell motility**	107	30	2	1	4E-02
**Energy metabolism**	393	246	8	11	4E-04
**Environmental adaptation**	161	36	3	2	7E-04
Folding sorting and degradation	571	274	11	12	4E-01
Lipid metabolism	229	124	4	5	1E-01
Membrane transport	75	20	1	1	6E-02
**Metabolism of other amino acids**	129	82	3	4	2E-02
**Nucleotide metabolism**	297	99	6	4	2E-02
**Replication and repair**	156	45	3	2	2E-02
**Signal transduction**	583	154	11	7	3E-07
Transport and catabolism	356	129	7	6	6E-02
**Translation**	559	315	11	14	6E-03
**Transcription**	264	155	5	7	2E-02

Boldface, subcategories that are significantly over-or under-represented in KN in comparison to GC-free; Number of proteins, total number of proteins in each subcategory; % of total, the relative contribution of each subcategory to the total number of proteins assigned KO numbers by KEGG; *P*-value adjusted, *P*-value adjusted by false discovery rate

To evaluate the effects of sequence redundancy (which might reflect true variation such isoforms, paralogs, and alleles, variation due to assembly errors, or both) we reduced the number of proteins in the GC and KN datasets so that KO numbers were represented only once per library (Additional file 2: Table S2). Although this stringent approach is most likely an unrealistic representation of transcriptome variation, any continued differences observed in transcript abundance between GC and KN datasets should bolster support for our previous protein count results. Some KEGG categories continued to show significant differences in transcript abundance between KN and GC with KN still having significantly more transcripts for energy metabolism than GC (Additional file 2: Table S2). Thus, there are real differences in protein abundance and diversity between KN and GC regardless of whether assembly quality influenced protein redundancy.

### Read count results

**Fig. 2:**
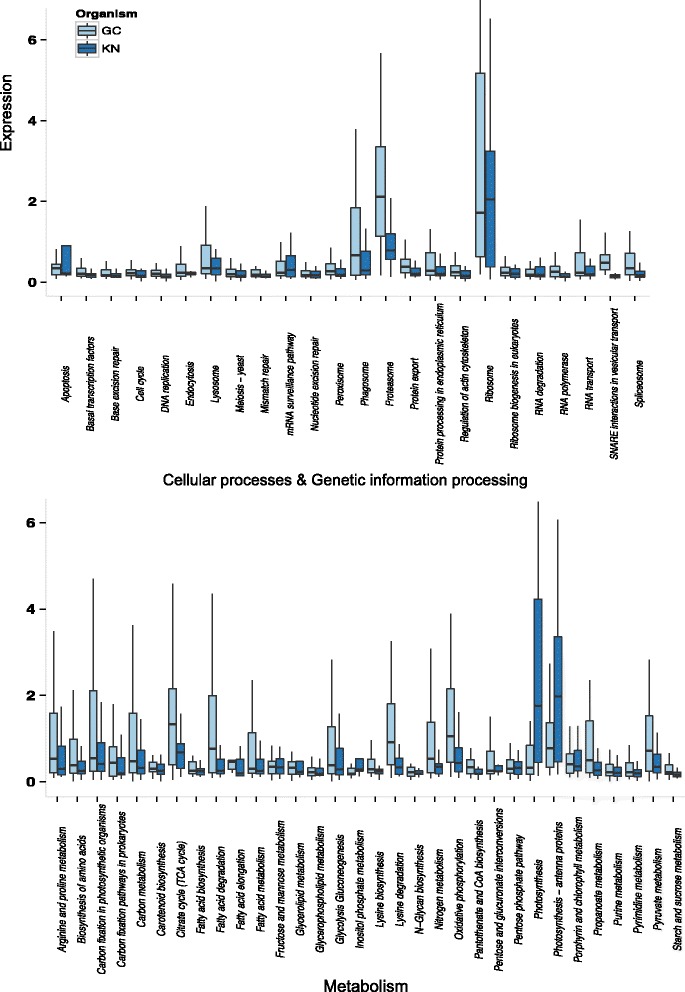
Boxplot of expression results for various pathways involved in cellular processing, genetic information processing, and metabolism. Expression levels equate to the number of reads per base pair for genes within each pathway. Outliers are not shown

We compared normalized gene expression differences between KN and GC for 21 KEGG subcategories and 89 pathways within those subcategories (Additional file 1: Table S1). Nearly all pathways exhibited decreased expression levels in KN in comparison to GC (Fig. 2) with 31 showing significant decreases (Additional file 3: Table S3). In contrast to the protein count results, read count analyses revealed significant decreases in transcription and amino acid metabolism expression for KN (Additional file 3: Table S3). While a greater proportion of amino acid metabolism and transcription proteins are present in KN, the overexpression of a few of these genes in each category could increase the average expression for GC. Additionally, KN could produce a greater diversity of proteins at lower expression levels than GC.

Only four metabolic pathways revealed significant increases in expression in KN: 1) cysteine and methionine metabolism, 2) inositol phosphate metabolism, 3) photosynthesis, and 4) photosynthesis—antennae proteins (Additional file 3: Table S3; Fig. 2). KN also showed an elevated average expression for the following pathways: porphyrin and chlorophyll metabolism, and carbon fixation in photosynthetic organisms, although none were significantly different from GC (Additional file 3: Table S3; Fig. 2). Most pathways involved in C metabolism, including glycolysis-gluconeogenesis, fructose-mannose metabolism, and lipid metabolism pathways, revealed similar expression levels in GC and KN (Additional file 3: Table S3; Fig. 2).

### Bioconductor analysis

Of the 3490 RBBH genes identified, DESeq detected 310 (120 were assigned KO numbers) genes with log2FC over-expression and 364 (188 were assigned KO numbers) with log2FC under-expression for KN in comparison to GC. For simplification, we report detailed results for changes in gene expression for DESeq analyses only as DESeq and edgeR produced similar findings. The majority of log2FC over-expressed genes (72.5 %) grouped to metabolic pathways (Additional file 4: Table S4) while only half of the log2FC under-expressed genes derived from this category (Additional file 5: Table S5). Of the thirteen reciprocal RBBH genes associated with the light-harvesting complex, all showed overexpression in KN with nine showing log2FC over-expression (Additional file 4: Table S4). These light harvesting complex I chlorophyll a/b binding proteins (LHCA1 and LHCA4) were also over-represented in transcript abundance analyses and contribute to the significantly increased expression of the light harvesting pathway (Fig. 2; Additional file 3: Table S3). All seven RBBH genes that coded for various photosystem I and II proteins or proteins associated with the photosystem demonstrated a log2fold increase in expression in KN as well (Additional file 4: Table S4). No genes related to photosynthesis appeared in the log2FC down-regulated category (Additional file 5: Table S5). The increased expression of light-harvesting and photosystem components in KN supports our previous analyses that detected significant changes in photosynthetic capacity between enslaved nuclei and free-living cells. Holistically, over-expressed genes in the energy metabolism category coded for proteins involved in carbon metabolism, photosynthesis, and carbon fixation (Additional file 4: Table S4) while under-expressed genes in this category coded for proteins that participated in oxidative phosphorylation and nitrogen metabolism (Additional file 5: Table S5).

Log2FC over- and under-expressed genes showed striking differences in other KEGG categories as well. Log2FC over-expressed genes assigned to the pathways for metabolism of cofactors and vitamins and the metabolism of terpenoids and polyketides coded for proteins with roles in oxidative stress management, including carotenoid biosynthesis and porphyrin and chlorophyll biosynthesis (Table 2). In fact, only one of the nine transcripts for carotenoid biosynthesis included in the DESeq analysis showed down-regulation (Table 2). The transcripts for the porphyrin and chlorophyll biosynthesis pathway displayed a signature of over-expression, with 79 % increasing expression by log2FC or more (Table 2). In contrast, DESeq identified no log2fold under-expressed genes for the carotenoid biosynthesis pathway (Additional file 5: Table S5). Overall, DESeq analyses support the read count results for up-regulation of photosynthetic pathways and highlight the increased expression of additional pathways related to photosynthesis, such as pigment production and oxidative stress reduction. Despite the lack of replication to account for biological variability, the differences between over and under-expressed gene categories suggest that real changes occur as the kleptokaryon becomes temporarily incorporated into the ciliate cell. However, we emphasize that these results provide insight into only one time period during the dynamic interplay between host and sequestered organelles and that additional experiments with replicates at various sampling points are necessary for strengthening support for our findings.

**Table 2:** Log2FC for genes belonging to select KEGG pathways relating to oxidative stress reduction and/or pigment production

KO	KN id	log2FC	KO description	Pathway
K01885	46,213	2.97	EARS, gltX; glutamyl-tRNA synthetase	Porphyrin & chlorophyll metabolism
K02492	12,431	3.72	hemA; glutamyl-tRNA reductase	Porphryin & chlorophyll metabolism
K01845	20,139	1.64	hemL; glutamate-1-semialdehyde 2,1-aminomutase	Porphryin & chlorophyll metabolism
K01698	9382	1.49	hemB, ALAD; porphobilinogen synthase	Porphryin & chlorophyll metabolism
K01749	22,375	1.43	hemC, HMBS; hydroxymethylbilane synthase	Porphryin & chlorophyll metabolism
K00589	15,072	−0.01	MET1; uroporphyrin-III C-methyltransferase	Porphryin & chlorophyll metabolism
K01599	31,220	3.08	hemE, UROD; uroporphyrinogen decarboxylase	Porphryin & chlorophyll metabolism
K01599	51300	2.25	hemE, UROD; uroporphyrinogen decarboxylase	Porphryin & chlorophyll metabolism
K00228	21674	2.17	CPOX, hemF; coproporphyrinogen III oxidase	Porphryin & chlorophyll metabolism
K00231	43719	1.87	PPOX, hemY; oxygen-dependent protoporphyrinogen oxidase	Porphryin & chlorophyll metabolism
K01772	15887	1.95	hemH, FECH; ferrochelatase	Porphryin & chlorophyll metabolism
K01772	18091	0.18	hemH, FECH; ferrochelatase	Porphryin & chlorophyll metabolism
K01772	19087	−0.72	hemH, FECH; ferrochelatase	Porphryin & chlorophyll metabolism
K02257	21293	0.08	COX10; protoheme IX farnesyltransferase	Porphryin & chlorophyll metabolism
K02259	1501	1.36	COX15; cytochrome c oxidase assembly protein subunit 15	Porphryin & chlorophyll metabolism
K03403	43039	3.56	chlH, bchH; magnesium chelatase subunit H	Porphryin & chlorophyll metabolism
K03403	15250	1.06	chlH, bchH; magnesium chelatase subunit H	Porphryin & chlorophyll metabolism
K03404	17580	2.21	chlD, bchD; magnesium chelatase subunit D	Porphryin & chlorophyll metabolism
K03428	10622	1.92	E2.1.1.11, chlM, bchM; magnesium-protoporphyrin O-methyltransferase	Porphryin & chlorophyll metabolism
K00218	28240	0.38	E1.3.1.33, por; protochlorophyllide reductase	Porphryin & chlorophyll metabolism
K00218	45156	0.42	E1.3.1.33, por; protochlorophyllide reductase	Porphryin & chlorophyll metabolism
K04040	21874	1.16	chlG, bchG; chlorophyll synthase	Porphryin & chlorophyll metabolism
K13606	65256	2.55	NOL, NYC1; chlorophyll(ide) b reductase	Porphryin & chlorophyll metabolism
K10960	19661	1.82	chlP, bchP; geranylgeranyl reductase	Porphryin & chlorophyll metabolism
K00510	28325	1.76	HMOX, hmuO, ho; heme oxygenase	Porphryin & chlorophyll metabolism
K00510	67894	2.01	HMOX, hmuO, ho; heme oxygenase	Porphryin & chlorophyll metabolism
K05370	8686	0.38	pebB; phycoerythrobilin:ferredoxin oxidoreductase	Porphryin & chlorophyll metabolism
K01764	44227	1.23	E4.4.1.17; cytochrome c heme-lyase	Porphryin & chlorophyll metabolism
K02291	46495	0.71	crtB; phytoene synthase	Carotenoid biosynthesis
K02291	41294	2.26	crtB; phytoene synthase	Carotenoid biosynthesis
K02293	16608	0.89	PDS, crtP; 15-cis-phytoene desaturase	Carotenoid biosynthesis
K02293	9496	2.23	PDS, crtP; 15-cis-phytoene desaturase	Carotenoid biosynthesis
K06443	39499	3.11	lcyB, crtL1, crtY; lycopene beta-cyclase	Carotenoid biosynthesis
K14606	6096	1.86	cruP; lycopene cyclase CruP	Carotenoid biosynthesis
K09837	28908	−3.68	LUT1, CYP97C1; carotene epsilon-monooxygenase	Carotenoid biosynthesis
K09838	40045	2.58	ZEP, ABA1; zeaxanthin epoxidase	Carotenoid biosynthesis

KO, KEGG orthology number assigned to each protein; KN id, MMETSP identification number for kleptokaryon proteins

**Fig. 3:**
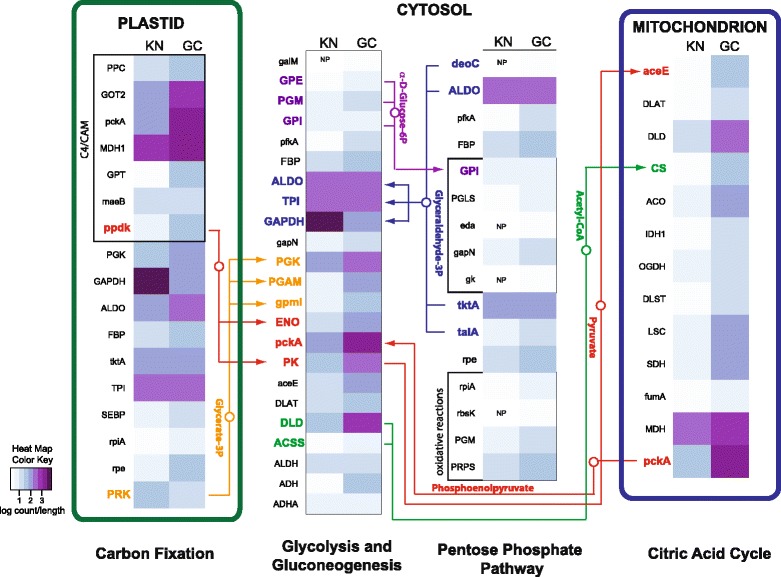
Carbon metabolism pathway mapping. A comparison of presence/absence and expression levels for genes involved in carbon metabolism for GC and KN. Expression values are log transformed. NP; not present

**Fig. 4:**
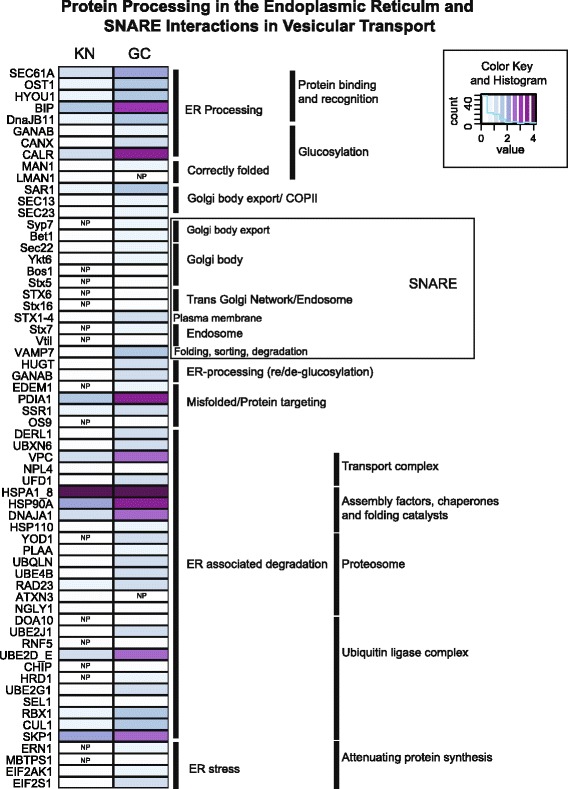
Protein processing and transport mapping. A comparison of presence/absence and expression levels for genes involved in protein processing in the endoplasmic reticulum and SNARE interactions in vesicular transport for KN and GC. Expression values are log transformed. NP; not present

To highlight some pathways in more detail, we examined the presence/absence patterns of transcripts in combination with their expression levels for select pathways. In this analysis, reads of all transcripts that represented the same KO number were combined and normalized by transcript length. KN and GC showed a high degree of similarity for carbon fixation and glycolysis/gluconeogenesis in central C-metabolism, which suggests intact connectivity of these cryptophyte pathways in *M. rubrum* (Fig. 3). In contrast, the KN almost completely lacked transcripts present in GC that were associated with exon splicing and 5’ capping during post-transcriptional modification (Additional file 6: Figure S1) as well as ER protein processing and vesicular transport (Fig. 4).

## Discussion

### Clues to the long-term maintenance of functional plastids

Despite their ubiquity in marine microbial food webs, few molecular studies of acquired phototrophic organisms are available and therefore relatively few conclusions can be made regarding common evolutionary strategies. *Mesodinium rubrum* is one of the most abundant and ecologically important acquired phototrophs [1]. The success of this ciliate can be largely attributed to its atypical ability to retain a functional cryptophyte nucleus, which it uses to control stolen organelles. Here we provide evidence that global transcription of *M. rubrum* is dominated by expression from the sequestered *Geminigera cryophila* (GC) nucleus (the kleptokaryon, or KN) for at least one sampling point during the life of an *M. rubrum* culture. While previous research on the same strain of *M. rubrum* demonstrated that the sequestered KN remains transcriptionally active for >30 days and persists without dividing [6], we detail for the first time overall changes that occurred to the GC transcriptome after its transition to the KN.

Carbohydrate metabolism pathways of the KN in *M. rubrum,* including, glycolysis/gluconeogenesis, fructose and mannose metabolism, carbon fixation, and photosynthesis (Figs. 2 and 3), increased or maintained expression levels in comparison to free living *Geminigera cryophila*, which supports the idea that *M. rubrum* sequesters not just the organelle machinery of its prey, but also the anabolic potential of sequestered organelles as well. These results help to explain how *M. rubrum* satisfies >90 % of its C needs via photosynthesis [8, 9]. We found that the KN of *M. rubrum* expresses a variety of genes required for pigment biosynthesis and plastid division (Fig. 2 and Table 2), providing *M. rubrum* with a mechanism for maintaining a constant plastid cell quota during cell division [6, 36], photoacclimating, repairing damaged photosystem proteins [37], and retaining function of its plastids for long periods (months) while starving [8]. Expression levels of select genes encoded within the sequestered plastid and nucleomorph were previously shown to be at their highest when the KN was present [6]. However, the growth of *M. rubrum* and the division of its plastids continues for several weeks after most members of the population have lost their KN [6]. Furthermore, the plastid-encoded *psbA* gene remains transcriptionally active in *M. rubrum* for at least 100 days [6]. In a temperate strain of the ciliate, plastids and plastid genes have also been shown to persist for long periods - up to 80 days [38]. The over-expression of KN-encoded genes involved in photosynthesis, including those coding for photosystem, light-harvesting, and electron transport chain proteins (Fig. 2, Additional file 3: Table S3 and Additional file 4: Table S4) might provide a protein reservoir that enables the longer retention of functional plastids in *M. rubrum* in comparison to other acquired phototrophs. The up-regulation of these genes by the KN might also be necessary to adequately service the number of plastids that *M. rubrum* retains. While cryptophytes typically harbor one plastid per cell, *M. rubrum* maintains up to 36 cell per cell [5] with one KN, effectively increasing the demand for plastid-destined proteins produced by this organelle.

In *M. rubrum*, the concomitant increased expression of KN genes coding for proteins involved in the production of ROS scavengers and accessory pigments likely all play key roles in reducing the damage incurred by up-regulated photosynthetic activities. This strategy seems to be employed by other kleptoplastidic ciliates as well. For example, the ciliate *Paramecium bursaria* decreased expression of a key oxidative stress gene, glutathione S-transferase, when harboring *Chlorella* endosymbionts, which suggests that its endosymbionts have roles in ROS protection [17]. Similarly, physiological studies of aposymbiotic and symbiotic *P. busaria* demonstrated that the presence of *Chlorella* symbionts increased host antioxidant capacity [39]. *S. rassoulzadegani* appears to enhance its own photo-oxidative protection measures by expressing a transcript for a Nec3 ascorbic acid recycling enzyme that was not detected in the transcriptome of its heterotrophic relative, *Strombidinopsis* sp. [16]. Thus our results further underscore the important role of controlling reactive oxygen species (ROS) by acquired phototrophs.

### Transcriptional changes suggest regulation and chimerism

Because sequestered organelles are no longer part of an intact cell (i.e. the ciliate does not retain the cell membrane, flagellar apparatus, and most likely other portions of sequestered cryptophytes [40, 41]), a reduction in gene expression for many pathways would be expected and is supported by our results. However, the down-regulation of KN ribosomal protein production, the mRNA surveillance pathway, ER protein processing and vesicular transport are surprising given that the ciliate maintains a high capacity for phototrophy [8] and overexpresses plastid-targeted genes. These results suggest that the ciliate host subsumes some of the responsibilities for KN transcript modification as well as protein production, modification, and distribution to sequestered organelle complexes.

The fact that up to 14 % of nuclear-encoded genes in *Arabidopsis thaliana* produce proteins that are targeted to the plastid (The Arabidopsis Genome Initiative, 2000) underscores the importance of protein transport for the survival of these organelles. Host participation in protein transport and targeting to symbiotic organelles is thought to be one of the major evolutionary hurdles to the stable acquisition of an organelle [42, 43]. Primary plastids rely on transit peptides to direct precursor molecules (unfolded proteins destined for the chloroplast) to TIC/TOC protein complexes embedded within the inner and outer plastid membranes and translocate precursors from the cytosol into the plastid [44]. However, alternative importation routes exist, including one that involves vesicular transport from the ER to the plastid and is hypothesized to be the ancestral mechanism for protein trafficking to this organelle [43, 45]. In fact, the amoeboid protist *Paulinella chromatophora* routes proteins targeted to its “in-progress” primary plastid, or chromatophore, through the Golgi apparatus [46]. For organisms with secondary or tertiary plastids, transportation and importation of proteins to the plastid is even more complex and requires translocation into the ER lumen first [47, 48]. In *M. rubrum*, KN transcription of ER proteins involved in translocation as well as components of the Golgi complex, endosomes, and SNAREs that might deliver precursors to sequestered plastids is minimal to essentially non-existent (Figs. 2 and 4), which suggests that protein trafficking relies on the host ER system instead. Thus our findings are reminiscent of previous hypotheses regarding the role of the endomembrane system in the initial control and integration of the photosynthetic endosymbiont into the host cell.

Host communication with sequestered organelles might also be facilitated by the arrangement of these organelles within the ciliate. In general, *M. rubrum* may harbor ~4 to over 36 organelle complexes, each surrounded by a single membrane containing a plastid, a mitochondrion, cytoplasm, other organelles of cryptophyte origin, and a single cryptophyte nucleus (if present) [3, 41]. When present, the single KN is surrounded by a separate membrane that may include cryptophyte cytoplasm and mitochondria [6, 49]. The chimeric nature of host-symbiont organelles is also apparent through application of dual-labeled SSU rRNA fluorescence *in situ* hybridization probes, which indicates a high degree of spatial heterogeneity in transcript targeting of host and KN products (Fig. 1) [6]. Our transcriptome data mirror this structural complexity, and suggest that the interwoven network of acquired organelles represents an initial mechanism of metabolic integration, adaptation, and perhaps stable acquisition. While we do not present direct evidence for a ciliate derived mechanism of protein transport and symbiotic organelle regulation, the conspicuous absence of KN transcripts for these pathways and the organizational complexity of acquired organelles, are both consistent with this notion.

Interestingly, KN metabolic pathways did not demonstrate the same trend as pathways associated with genetic and cellular functions. For example, proteins related to overall carbohydrate and amino acid metabolism increased significantly in abundance in the KN (Table 1) with several amino acid, sugar, carbohydrate, and lipid metabolism/biosynthesis pathways in KN and GC remaining at equivalent expression levels (Fig. 2). These results support earlier observations of Johnson et al. (2006), who found photosynthetically fixed ^14^C was highly incorporated into host lipid and protein metabolic pools [36]. The striking up-regulation of photosynthetic abilities highlights the advantages of maintaining both cryptophyte nucleus and plastid organelles and suggests that their collective role and primary responsibility is to service the energy needs of the ciliate host. Although we lack replication to account for biological variation, our results support several previous analyses performed with this ciliate strain, which showed increases in production of chlorophyll *a* as well as increases in photosynthesis, growth rates, and plastid division in *M. rubrum* cultures fed with *G. cryophila* versus unfed controls [4, 8]. Our expression analyses are also consistent with previously performed qRT-PCR experiments that quantified over-expression of light-harvesting and photosystem complex genes, with expression levels up to tenfold higher for these genes than for *M. rubrum* housekeeping genes [6]. Similarly, successful PCR amplification of two plastid genes from a temperate strain of *M. rubrum* still occurred even after 80 days of starvation [38].

### M. rubrum as a model system for exploring prerequisites to organelle evolution

The evolution of the primary plastid from a cyanobacterial ancestor that was engulfed by a phagotrophic eukaryote [50] and the subsequent lateral spread of plastids through the eukaryotic tree of life via secondary [51] and tertiary endosymbioses [52] represent major drivers of eukaryotic innovation and diversification [48, 53].

The endosymbiotic acquisition of secondary and tertiary plastids involved massive gene transfer events from algal endosymbiont to host, the development transit sequences for targeting horizontally-transferred genes back to the plastid, and the evolution of transport complexes beyond the TIC/TOC translocons in primary plastids that allow proteins to cross up to five plastid membranes [42, 47]. However, other mechanisms required to transform an endosymbiont into an organelle remain mostly relegated to speculation. Additionally, the under-representation of protists in genome projects, the complicated history of protist lineages that diversified due to serial endosymbiotic events, and the often relatively close phylogenetic relationship between eukaryotic host and algal endosymbiont confound our abilities to disentangle the historical events that led to the evolution of secondary and tertiary plastids.

We propose that *M. rubrum* represents a model organism for identifying early adaptations to obligate phototrophy and potential prerequisites to stable plastid integration. Furthermore, while ciliates belong to the SAR supergroup (stramenopiles, alveolates, and rhizarians, which are comprised of several lineages with secondary and tertiary plastids) and contain a small subset of genes suggesting a potential photosynthetic ancestry [54], no members have functional or remnant plastids. Thus, *M. rubrum* provides a less complicated system to examine the symbiont–to-organelle transition.

## Conclusions

Our preliminary analyses into the molecular interactions between ciliate host and sequestered nucleus reveal dramatic increases in expression of photosynthetically related pathways in the KN with concomitant decreases in most cellular and genetic pathways. Pathways involved in protein import and export via the endomembrane system also showed significant decline in the KN. Collectively, our results suggest that *M. rubrum* maintains the KN of its cryptophyte prey to ensure plastid productivity while possibly exerting a measure of control over photosynthetic output via importation/exportation mechanisms. Further expression studies over various time periods and environmental conditions will clarify the general contribution of the KN to the ciliate transcriptome. Although *M. rubrum* cannot be cultured in the absence of its sequestered plastids, a comparison between the transcriptomes of *M. rubrum* and a closely related heterotroph (i.e. *Mesodinium pulex*) might help to elucidate compensatory changes that occur in the host as it adjusts to a mixotrophic lifestyle.

## Additional files

Additional file 1: Table S1. KEGG Categories evaluated under protein and read count tests. 1. Metabolism, 2. Genetic information Processing, 3. Environmental information processing, and 4. Cellular Processes represent broad KEGG categories. Subcategories in bold (1.0–4.3) were subject to protein count analyses while italicized pathways within these subcateogries were analyzed by the read count method. (XLSX 17 kb) Additional file 2: Table S2. Reduced protein count analyses for KEGG categories. Boldface, subcategories that are significantly over-or under-represented in KN in comparison to GC; Number of KOs; The number of KOs that belong to each subcategory where only one protein representative for each KO was included in the analysis; % of total, the relative contribution of each subcategory to the total number of KOs assigned by KEGG. (XLSX 10 kb) Additional file 3: Table S3. Differential expression of KEGG pathways between GC and KN. GC and KN, mean expression values associated with each pathway calculated from the total number of reads per base pair (normalized read counts for each gene); P adj., *P* value as determined from Mann-Whitney tests and adjusted with a Benjamini and Hochberg false discovery rate < 0.05 to identify significant differences in expression between GC and KN for each pathway; General cateogry, the more inclusive category to which each pathway belongs; boldface represents pathways with significant differences in expression between GC and KN. (XLSX 26 kb) Additional file 4: Table S4. Description and classification of DESeq log2FC over-expressed genes in KN. Genes of note are in bold with parenthetical notation indicating the pathway to which they belong. (XLSX 37 kb) Additional file 5: Table S5. Description and classification of DESeq log2FC under-expressed genes in KN. Genes of note are in bold with parenthetical notation indicating the pathway to which they belong. Genes were assigned to their structural or enzymatic function (for example, “Chromosome”, “Cytoskeleton Proteins”, “Peptidases”, and “Ubiquitin System”) when they returned associations with categories not relevant to this system (for example, Human Diseases and Organismal Processes). (XLSX 48 kb) Additional file 6: Figure S1. mRNA surveillance pathway mapping. A comparison of presence/absence and expression levels for genes involved in mRNA surveillance pathways for KN and GC. Expression levels are log transformed. (PDF 179 kb)
