# Supplementary figures and images for: Insights into transcriptional changes that accompany organelle sequestration from the stolen nucleus of Mesodinium rubrum
Source: BMC Genomics. 2015 Oct 16;16:805. doi: 10.1186/s12864-015-2052-9 (PMC4609049; doi:10.1186/s12864-015-2052-9)

# mRNA Surveillance Pathway

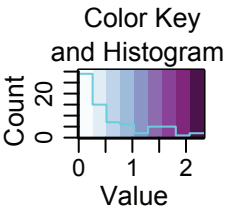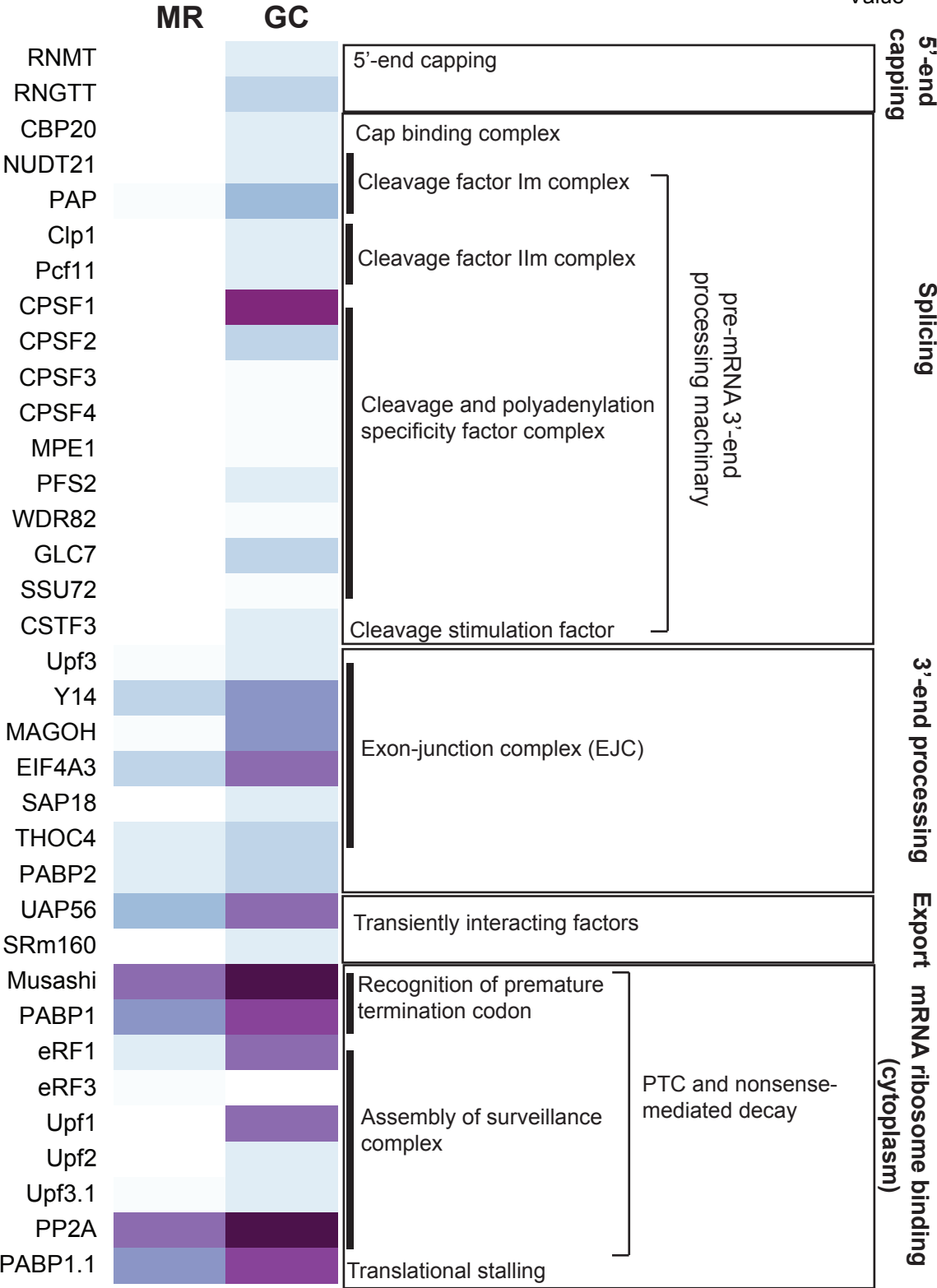

Supplement: Additional file 6: Figure S1. — mRNA surveillance pathway mapping. A comparison of presence/absence and expression levels for genes involved in mRNA surveillance pathways for KN and GC. Expression levels are log transformed. (PDF 179 kb) [file 12864_2015_2052_MOESM6_ESM.pdf]
